# A Comparative Study of Systems Pharmacology and Gene Chip Technology for Predicting Targets of a Traditional Chinese Medicine Formula in Primary Liver Cancer Treatment

**DOI:** 10.3389/fphar.2022.768862

**Published:** 2022-03-02

**Authors:** Songzhe Li, Yang Sun, Yue Sun

**Affiliations:** Department of Biology, College of Basic Medicine, Heilongjiang University of Chinese Medicine, Harbin, China

**Keywords:** ZZXJT, systems pharmacology, gene chip, molecular docking, liver cancer, drug target prediction comparing TCM target prediction models 2

## Abstract

**Background:** The systems pharmacology approach is a target prediction model for traditional Chinese medicine and has been used increasingly in recent years. However, the accuracy of this model to other prediction models is yet to be established.

**Objective** *:* To compare the systems pharmacology modelwithexperimental gene chip technology by using these models to predict targets of a traditional Chinese medicine formulain the treatment of primary liver cancer.

**Methods:** Systems pharmacology and gene chip target predictions were performed for the traditional Chinese medicine formula *ZhenzhuXiaojiTang* (ZZXJT). A third square alignment was performed with molecular docking.

**Results:** Identification of systems pharmacology accounted for 17% of targets, whilegene chip-predicted outcomes accounted for 19%.Molecular docking showed that the top ten targets (excludingcommon targets) of the system pharmacology model had better binding free energies than the gene chip model using twocommon targets as a benchmark. For both models, the core drugs predictions were more consistent than the core small molecules predictions.

**Conclusion:**In this study, the identified targets of systems pharmacology weredissimilar to those identified by gene chip technology; whereas the core drug and small molecule predictions were similar.

## 1 Introduction

Traditional Chinese medicine (TCM) has a long history of clinical application in China andforms a complete and independent system of diagnosis and treatment. TCM is efficaciousin the treatment of many diseases, including cancer (Fang et al., 2017). In the treatment of cancer, TCMnot only shows great potential as a means of medical intervention (Yan et al., 2017) but may also be modified according to patient comorbidities. In TCM, sovereign drugs (drugs used to treat the primary disease) may be used together with adjuvant drugs (drugsused to improve the efficacy of the sovereign drugor totreat other symptoms), thus alleviating pain and improving quality of life. TCM usually follows the principle of multiple-herbformulasduring clinical application. In the treatment of a single disease, the core combination of drugs is difficult to change; however, adjuvant drugs may be added to increasethe efficacy of the drug combination or totreat secondary diseases. At Heilongjiang University of Chinese Medicine, our experimental group graduallyformed a unique drug regimen for the treatment of primary liver cancer, by diagnosing and treating primary liver cancer with a large collection of herbs, and then observing the clinical efficacy of these herbs in patients. Using this clinical experience, we finally identified five crucial herbs, including*Ligustrum* (NZZ), *Curcumaerhizoma* (EZ), *Prunella vulgaris* (XKC), *Hedyotisdiffusa* (BH), and *Glycyrrhizae radix* (GC). According to theory, TCM formulasconsist of sovereign, adjuvant, assistant, and guide drugs. We applied this principle and assignedNZZ and EZasthe sovereign drugs, XKC and BH asthe adjuvant drugs, and GC as the assistant and guide drug. These five drugs made upthe formula for treating primary liver cancer and we named it*ZhenzhuXiaojiTang* (ZZXJT). We initially administered ZZXJT toa murineH22 hepatocarcinomamodel. TheZZXJT group showedH22 cell degeneration and necrosis and the number of blood vessels was reduced. Additionally, many autophagosomes in H22 cells were observed by transmission electron microscopy. Thesefindings revealed that ZZXJT may induce programmed H22 cell death and inhibit primary liver cancer development (Sun et al., 2017).

TCM uses a multi-component and multi-target approach (Pei et al., 2013), which can be both advantageous and disadvantageous in modern medical research. Advantages includesingle drugsexhibiting multiple mechanisms (Miao et al., 2020; El-Zayat et al., 2021), flexibility of multi-drug treatment (Ren et al., 2020; Zhang et al., 2021), and pairing of drugsto improve drug efficacy (Luo C. H. et al., 2020). However, the disadvantages are consequences of these advantages. A single drug comprisesnumerous compounds which in turn comprise different small molecules that vary in their pharmacological activity; hence, targets are difficult to identify in experimental studies. For example, *HoupuDahuang* decoction, *HoupuSanwu* decoction, and *Xiaochengqi* decoction include the herbs*Mangnolia officinalis, Rheum palmatum*, and *Citrus aurantium* but the pharmacological effects and indications of the three formulas differ. *HoupuDahuang* decoction is mainly used to treat cough and exudative pleurisy, while the *HoupuSanwu* decoction is primarily used to treat paralytic ileus and *Xiao Chengqi* decoction is mainly used to treat adherent ileus, chronic gastritis, and intestinal paralysis (Kou et al., 2004). It is therefore challenging to regulate results and form a research strategy when core combinations of drugs are studied. Accurate TCM target prediction is crucial due to the rapid development of TCM ingredients in the post-genomic era. The systems pharmacology approach and gene chip technology provide effective target prediction methods for TCM laboratory research, thereby guiding research direction. These prediction models play important roles in drug development and research (Cooke et al., 2009; Boezio et al., 2017; Luo T. T. et al., 2020), as indicated by the increased number of studies in recent years on systems pharmacology particularly (Jiao et al., 2021). However, since the methods and principles of the two prediction models are very different, predicted results heavily influence the research direction.

Systems pharmacologymakes use ofa data platformthat predicts drug-target interactions based on network analyses from previously published research data (Berger et al., 2010). In gene chip technology, a large number of known gene sequences are immobilised and hybridised onto a glass chip, allowing for large-scale prediction (Yanagawa et al., 2005). Molecular docking is used as an auxiliary model for systems pharmacology and gene chip technology. Docking data corroborate the results of the prediction models to check reliability (Li et al., 2021). Although systems pharmacology is cost-effective and saves time compared with the experimental high-throughput screening used in gene chip technology (Yuan et al., 2017), the differences in predictions between the two models have rarely been reported, especially in the research of TCM formulas. This study, therefore, aims toguide the future selection of methods for TCM target prediction.

## 2 Materials and Methods

### 2.1 Systems Pharmacology Model of ZZXJT

#### 2.1.1 Screening of Active Ingredients and Related Targets ofZZXJT

We searched for the active ingredients of thefive herbs, EZ, NZZ, BH, XKC, and GConthe traditional Chinesemedicine systems pharmacology database andanalysis platform (TCMSP) (Ru et al., 2014). We screenedthese active ingredients by specifyingtwo ADME-related properties as the screening criteria, namely, oral bioavailability (OB) ≥ 30% and drug-likeness (DL) ≥ 0.18. Active ingredients without targets were removed and we integratedthe identified active ingredient targets. After determining the target protein information, the collected target information was unified in UniProt protein database^1^, to normalise it (UniProt Consortium, 2021). We then constructed the “drug active ingredient-target” network.

#### 2.1.2 Screening for Liver Cancer Targets

Using “liver cancer” as the keyword, potential liver cancer-related targets were mined from the OMIM database^2^ and GeneCards database^3^ (downloaded December 2020). Liver cancer-related genesidentified by the GeneCards database were filtered andonly results with relevance score ≥15 wereretained. Data collected from the two databases weremerged and denoted as Dataset 1.

#### 2.1.3 Construction and Analysis of “Drug Active Ingredient-Target” Network

To further clarify the mechanism and relevance of the interaction between ZZXJT and hepatocellular carcinoma (HCC), intersecting targets between ZZXJT (drug), and HCC (disease) were obtained by drawing Venn diagrams^4^. These intersecting targets were submitted to the STRING database^5^ (Szklarczyk et al., 2019) and a protein-protein interaction (PPI) network was constructed by setting “Organism” to “*Homo sapiens*” and confidence level to 0.4. This network was imported to Cytoscape software (version 3.70) and core potential proteins were analysed by adjustingvisualisation parameters according to the “combined degree” values of individual nodes. The functions of the target proteins involved in the biological process were described.

#### 2.1.4 Gene Enrichment Analysis

We selected Metascape database^6^ (Tripathi et al., 2015; Zhou et al., 2019) as the gene-list analysis portal because it is updated regularly and is data comprehensive. The relevant ZZXJTand HCC targets were entered into the Metascape database and screening criteria were set as follows: statistical difference at *p* < 0.01 and mode of analysisas custom analysis. The following analyses were then carried out: Kyoto Encyclopaedia of Genes and Genomes (KEGG) pathway analysis and Gene Ontology (GO). GO has three subdivisions that were conducted, namely, molecularfunction (MF), biologicalprocess (BP), and cellularcomponent (CC). KEGG pathway analysis yielded the top 20 results and BP, CC, and MF yielded the top 10 results. Results were displayed in bubble plots, created using ImageGP^7^.

### 2.2 Gene Chip Model of ZZXJT

#### 2.2.1 Cell Culture

The cells were routinely cultured in 25 cm^2^ culture flask in DMEM medium (Boster, #PYG0103) supplemented with 5% fetal bovine serum and 1% antibiotics (penicillin/streptomycin). When the cells placed under the culture flask about 70–80%, the original culture medium was discarded, and the culture flask was rinse with PBS gently. Then trypsinization was added in the flask. When the intercellular space of cells enlarged, fresh culture medium was put in the flask to finish trypsin digestion. After centrifugation, the culture medium was extracted carefully on the cell upper layer, and then the cells were made a single cell suspension by the fresh culture medium. The cells were cultured in a constant temperature (37°C) with a volume fraction 5% CO_2_.

#### 2.2.2 RNA Extraction and RNA Quality Control

Six 25 cm^2^ cell culture flasks of SMMC-7721 cells were cultured together for 24 h. Three flasks were used as the control group (sample numbersA12854, A12855, and A12856) and the remainder as the drug group, in which 20% ZZXJT-containing serum was added to each flask (sample numbersA12857, A12858, and A12859). RNA was extracted from samples using the TRIzol reagent (Invitrogen, United States), according to the manufacturer’s instructions. We measured the A260/A280 ratio using a NanoDrop 2000 spectrophotometer (Thermo Fisher Scientific, United States). The required reagents were then left at room temperature for 30 minand the sample and RNA ladder were placed on ice. A 550 µl RNA 6000 Nano gel matrix (Agilent Technologies, United States) was placed in a centrifuge tube and centrifuged at 1500 *g* for 10 min at room temperature. We then added 1 µl of dye to 65 µl of the gel, shook it well, and centrifugedit at 13000 *g* for 10 min at room temperature to make the gel-dye mix. Next, we added 9 µl of the gel-dye mix to the GN-genechipClariom™ S Array, human gene chip (catalogue number 902927, Affymetrix, United States) by pressingthe gel-dye mix into the gene chip capillary with a piston. 5 µl of RNA 6000 Nano maker (Agilent Technologies, United States)was added to the sample well and ladder well. Finally, we added 1 µl of denatured ladder into the Agilent 2,100 instrument (Agilent Technologies, United States) to assess the RNA integrity number (RIN) and the 28S/18S ratio. Data were analysed using the Agilent 2,100 Expert software. Quality control standards were as follows: A260/A280 ratio of 1.7–2.2, RIN≥7.0, and 28S/18S > 0.7.

#### 2.2.3 Gene Chip Preparation and Hybridization

The first and second strands of the cDNA were synthesised. Labelling cRNA was synthesised by *in vitro* transcription. The synthesised cRNAs were then purified and quantified. Single-stranded cDNA was synthesised in the second cycle. cRNA was hydrolyzed by RNase H and single-stranded cDNA remained. After the second cycle of single-stranded cDNA was purified, its concentration was measured. The purified ss-cDNA was transformed into dUTP residual fragments and broken DNA strands, which were covalently linked to biotin, Affymetrix proprietary DNA labelling reagent, to complete cDNA fragmentation and labelling, using. The gene chip was removed and hybridisation and washing were performed.

#### 2.2.4 Construction and Analysis of the “Drug Active Ingredient-Target” Network

The obtained gene chip microarray data were combined with Dataset 1, and a Venn diagram was drawn to determine the intersectinggene chip-predicted targets and HCC targets. The intersecting targets were submitted to the STRING database and the PPI network model was constructed by setting“Organism” to “Homo sapiens” and confidence level to 0.4. This network was imported to Cytoscape software (version 3.70) and core potential proteins were analysed by adjusting visualisation parameters according to the “Combined degree” values of individual nodes. The functions of the target proteins involved in the biological process were described.

#### 2.2.5 Gene Enrichment Analysis

The gene chip-predicted targets of ZZXJT and relevant targets of HCC were entered into the Metascape database. Screening criteria were set as follows: statistical difference as *p* < 0.01 and the analysis mode as custom analysis. KEGG pathway analysis and GO analysis at MF, BP, and CC levels, among others, were then performed.

#### 2.2.6 qRT-PCR Assay

The total RNA of drug group and control group was extracted with Trizol (Invitrogen, United States). Use Prime Script first Stand cDNA Synthesis Kit to reverse transcribe RNA into cDNA according to the instructions. Adopt SYBR pre-mixed ExTaq kit (Takara, Dalian, China), PCR amplification was conducted at 40 cycles of denaturation at 95°C for 30s, after pre-denaturation treatment at 95°C for 5 min, annealing treatment 58°C for 30 s and extension at 72°C for 30 s. The temperature at the end was 4°C. GAPDH was used as the internal reference gene to detect the relative expression levels of AKT, FOXO1, FOXO3, and FOXO4. The primers of these genes were displayed in Table 1. Each sample was repeated 3 times to ensure the accuracy of the data. The relative expression levels of these genes were calculated in accordance with 2^−∆∆ct^.

**TABLE 1:** Primer sequences of qRT-PCR.

Genes	Sequences
GAPDH	F: 5′-GGA​GCG​AGA​TCC​CTC​CAA​AAT-3′
	R:5′-GGCTGTTGTCATACTTCTCATGG-3′
AKT	F: 5′-GTC​ATC​GAA​CGC​ACC​TTC​CAT-3′
	R: 5′-AGC​TTC​AGG​TAC​TCA​AAC​TCG​T-3′
FOXO1	F: 5′-AAG​GAT​AAG​GGT​GAC​AGC​AAC​AG-3′
	R: 5′-TTG​CTG​TGT​AGG​GAC​AGA​TTA​TGA​C-3′
FOXO3	F: 5′-CTA​CGA​GTG​GAT​GGT​GCG​TT-3′
	R: 5′-TGC​CAG​TTC​CCT​CAT​TCT​GG-3′
FOXO4	F: 5′-CAC​TGT​GCC​AAT​TAG​GGG​GT-3′
	R: 5′-CTC​CCA​AAG​GCA​GGG​GTA​AG-3′

### 2.3 Identification Treatment of Two Models

The resulting sets of data from Section 2.1.3 and Section 2.2.3 were combined and a Venn diagram was drawn to determine intersection of predicted targets. The intersecting data were imported into the STRING database and the PPI network model was constructed by setting “Organism” to “Homo sapiens” and confidence level to 0.4. The resulting network was imported into Cytoscapesoftware (version 3.70) for analysis. The free proteins were identified by screening core potential proteins by adjusting the visualisation parameters according to the “Combined degree” values of individual nodes. We then performed KEGG pathway analysis using the Metascape database.

### 2.4 Molecular Docking Validation and Comparison

#### 2.4.1 Main Active Ingredients of ZZXJT

According to the TCM theory of sovereignand adjuvant drugs, the active ingredients of sovereigndrugs in Table 2 and the active ingredients shared by *Ligustrum* (NZZ) and*Curcumaerhizoma* (EZ) were integrated. The mol2 file corresponding to these active ingredients was downloaded from the TCMSP database for later use.

**TABLE 2:** Main active ingredients of ZZXJT.

Drug No	MOL ID	Main active ingredients	DL	OB%
BH1	MOL001659	Poriferasterol	0.76	43.8
BH2	MOL001670	2-methoxy-3-methyl-9,10-anthraquinone	0.21	37.8
EZ1	MOL000296	hederagenin	0.75	36.9
GC1	MOL001484	Inermine	0.54	75.2
GC2	MOL001792	DFV	0.18	32.8
GC3	MOL002311	Glycyrol	0.67	90.8
GC4	MOL000239	Jaranol	0.29	50.8
GC5	MOL002565	Medicarpin	0.34	49.2
GC6	MOL000354	isorhamnetin	0.31	49.6
GC7	MOL000359	sitosterol	0.75	36.9
GC8	MOL003656	Lupiwighteone	0.37	51.6
GC9	MOL003896	7-Methoxy-2-methyl isoflavone	0.2	42.6
GC10	MOL000392	formononetin	0.21	69.7
GC11	MOL000417	Calycosin	0.24	47.8
GC13	MOL004328	naringenin	0.21	59.3
GC14	MOL004805	(2S)-2-[4-hydroxy-3-(3-methylbut-2-enyl)phenyl]-8,8-dimethyl-2,3-dihydropyrano [2,3-f]chromen-4-one	0.72	31.8
GC15	MOL004806	euchrenone	0.57	30.3
GC16	MOL004808	glyasperin B	0.44	65.2
GC17	MOL004810	glyasperin F	0.54	75.8
GC18	MOL004811	Glyasperin C	0.4	45.6
GC19	MOL004814	Isotrifoliol	0.42	31.9
GC20	MOL004815	(E)-1-(2,4-dihydroxyphenyl)-3-(2,2-dimethylchromen-6-yl)prop-2-en-1-one	0.35	39.6
GC21	MOL004820	kanzonols W	0.52	50.5
GC22	MOL004824	(2S)-6-(2,4-dihydroxyphenyl)-2-(2-hydroxypropan-2-yl)-4-methoxy-2,3-dihydrofuro [3,2-g]chromen-7-one	0.63	60.3
GC23	MOL004827	Semilicoisoflavone B	0.55	48.8
GC24	MOL004828	Glepidotin A	0.35	44.7
GC25	MOL004829	Glepidotin B	0.34	64.5
GC26	MOL004833	Phaseolinisoflavan	0.45	32
GC27	MOL004835	Glypallichalcone	0.19	61.6
GC28	MOL004838	8-(6-hydroxy-2-benzofuranyl)-2,2-dimethyl-5-chromenol	0.38	58.4
GC29	MOL004841	Licochalcone B	0.19	76.8
GC30	MOL004848	licochalcone G	0.32	49.3
GC31	MOL004849	3-(2,4-dihydroxyphenyl)-8-(1,1-dimethylprop-2-enyl)-7-hydroxy-5-methoxy-coumarin	0.43	59.6
GC32	MOL004855	Licoricone	0.47	63.6
GC33	MOL004856	Gancaonin A	0.4	51.1
GC34	MOL004857	Gancaonin B	0.45	48.8
GC35	MOL004863	3-(3,4-dihydroxyphenyl)-5,7-dihydroxy-8-(3-methylbut-2-enyl)chromone	0.41	66.4
GC36	MOL004864	5,7-dihydroxy-3-(4-methoxyphenyl)-8-(3-methylbut-2-enyl)chromone	0.41	30.5
GC37	MOL004866	2-(3,4-dihydroxyphenyl)-5,7-dihydroxy-6-(3-methylbut-2-enyl)chromone	0.41	44.2
GC38	MOL004879	Glycyrin	0.47	52.6
GC39	MOL004882	Licocoumarone	0.36	33.2
GC40	MOL004883	Licoisoflavone	0.42	41.6
GC41	MOL004884	Licoisoflavone B	0.55	38.9
GC42	MOL004885	licoisoflavanone	0.54	52.5
GC43	MOL004891	shinpterocarpin	0.73	80.3
GC44	MOL004898	(E)-3-[3,4-dihydroxy-5-(3-methylbut-2-enyl)phenyl]-1-(2,4-dihydroxyphenyl)prop-2-en-1-one	0.31	46.3
GC45	MOL004903	liquiritin	0.74	65.7
GC46	MOL004904	licopyranocoumarin	0.65	80.4
GC47	MOL004907	Glyzaglabrin	0.35	61.1
GC48	MOL004908	Glabridin	0.47	53.3
GC49	MOL004910	Glabranin	0.31	52.9
GC50	MOL004911	Glabrene	0.44	46.3
GC51	MOL004912	Glabrone	0.5	52.5
GC52	MOL004913	1,3-dihydroxy-9-methoxy-6-benzofurano [3,2-c]chromenone	0.43	48.1
GC53	MOL004914	1,3-dihydroxy-8,9-dimethoxy-6-benzofurano [3,2-c]chromenone	0.53	62.9
GC54	MOL004915	Eurycarpin A	0.37	43.3
GC55	MOL004924	(-)-Medicocarpin	0.95	41
GC56	MOL004935	Sigmoidin-B	0.41	34.9
GC57	MOL004941	(2R)-7-hydroxy-2-(4-hydroxyphenyl)chroman-4-one	0.18	71.1
GC58	MOL004945	(2S)-7-hydroxy-2-(4-hydroxyphenyl)-8-(3-methylbut-2-enyl)chroman-4-one	0.32	36.6
GC59	MOL004948	Isoglycyrol	0.84	44.7
GC60	MOL004949	Isolicoflavonol	0.42	45.2
GC61	MOL004957	HMO	0.21	38.4
GC62	MOL004959	1-Methoxyphaseollidin	0.64	70
GC63	MOL004961	Quercetin der	0.33	46.5
GC64	MOL004966	3′-Hydroxy-4′-O-Methylglabridin	0.57	43.7
GC65	MOL000497	licochalcone a	0.29	40.8
GC66	MOL004974	3′-Methoxyglabridin	0.57	46.2
GC67	MOL004978	2-[(3R)-8,8-dimethyl-3,4-dihydro-2H-pyrano [6,5-f]chromen-3-yl]-5-methoxyphenol	0.52	36.2
GC68	MOL004980	Inflacoumarin A	0.33	39.7
GC69	MOL004985	icos-5-enoic acid	0.2	30.7
GC70	MOL004988	Kanzonol F	0.89	32.5
GC71	MOL004989	6-prenylated eriodictyol	0.41	39.2
GC72	MOL004990	7,2′,4′-trihydroxy-5-methoxy-3-arylcoumarin	0.27	83.7
GC73	MOL004991	7-Acetoxy-2-methylisoflavone	0.26	38.9
GC74	MOL004993	8-prenylated eriodictyol	0.4	53.8
GC75	MOL004996	gadelaidic acid	0.2	30.7
GC76	MOL000500	Vestitol	0.21	74.7
GC77	MOL005000	Gancaonin G	0.39	60.4
GC78	MOL005001	Gancaonin H	0.78	50.1
GC79	MOL005003	Licoagrocarpin	0.58	58.8
GC80	MOL005007	Glyasperins M	0.59	72.7
GC81	MOL005008	Glycyrrhiza flavonol A	0.6	41.3
GC82	MOL005012	Licoagroisoflavone	0.49	57.3
GC83	MOL005016	Odoratin	0.3	50
GC84	MOL005017	Phaseol	0.58	78.8
GC85	MOL005018	Xambioona	0.87	54.9
GC86	MOL005020	dehydroglyasperins C	0.37	53.8
NZZ1	MOL004576	taxifolin	0.27	57.8
NZZ2	MOL005147	LucidumosideD_qt	0.47	54.4
NZZ3	MOL005190	eriodictyol	0.24	71.8
NZZ4	MOL005212	Olitoriside_qt	0.78	103
XKC1	MOL004355	Spinasterol	0.76	43
XKC3	MOL004798	delphinidin	0.28	40.6
XKC4	MOL006767	Vulgaxanthin-I	0.26	56.1
XKC5	MOL006772	poriferasterol monoglucoside_qt	0.76	43.8
XKC6	MOL006774	stigmast-7-enol	0.75	37.4
XKC7	MOL000737	morin	0.27	46.2
A1	MOL000098	quercetin	0.28	46.4
A2	MOL000449	Stigmasterol	0.76	43.8
A3	MOL000359	beta-sitosterol	0.75	36.9
B1	MOL000422	kaempferol	0.24	41.9
C1	MOL000006	luteolin	0.25	36.2

#### 2.4.2 Selection of Docking Targets

We downloaded the protein crystal structures corresponding to the common target genes of the two models from the RCSB PDB database^8^, using the search criteria “Homo sapiens” and “protein”Thetarget gene proteins were then ranked by “Combined degree” values of the two sets of models. Crystal structures of the top tenwere selected (excluding the common target proteins) and the PDB numbers were recorded.

#### 2.4.3 Molecular Docking Software and Protocol

AutoDock Vina (version 1.5.6) was the software used for molecular docking (Trott and Olson, 2010). According to the AutoDock report, 78% of molecular target docking results had aroot mean square deviation (RMSD) < 2. RMSD <2 was considered feasible, therefore, molecular docking predictions conducted using AutoDock could be considered accurate. The collected small molecules and target proteins of ZZXJT were prepared by removing water molecules, adding hydrogen atoms, and setting semiflexible docking and blind docking methods as parameters. The remaining parameters were set to default values. Given that this study already covered experimental microarray prediction, molecular docking was used as a third-party reference model. The possibility of interfering conditions such as hydrogen bonds was therefore ignored andonly maximum binding energy was taken as a reference. The free energy results of the system pharmacology model, the gene chip model, and the common target gene proteins were then statistically aligned using SPSS 26.0 and the independent samples *t*-test method was used.

## 3 Results

### 3.1 Systems Pharmacology Model of ZZXJT

#### 3.1.1 Identified Active Ingredients and Targets of ZZXJT

**FIGURE 1:**
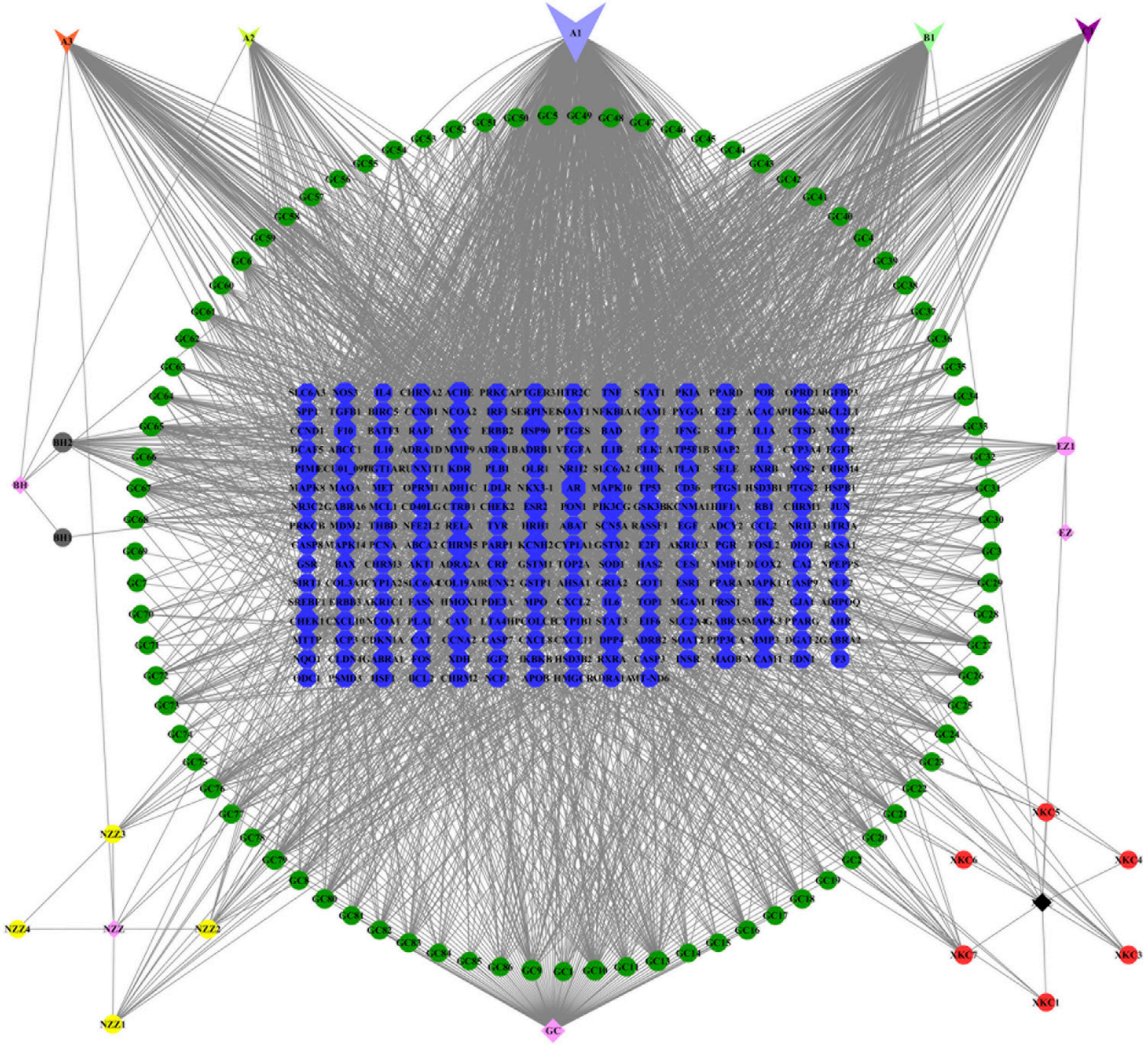
“ZZXJT active ingredient-target” network diagram.

A total of 126 chemical components were initially extracted from ZZXJT and 103 active components were identified after ADME screening and normalisation of the UniProt protein database, including poriferosterol, glycyrol, and hederagenin. As shown in Table 2, these small molecules, numbered A1 (BH, GC, XKC, and NZZ), A2 (BH, XKC), A3 (BH, XKC, and NZZ), B1 (GC, XKC, and NZZ), and C1 (XKC,NZZ) are common to many drugs. After merging and removing duplicates, the number of ingredient targets was 235. Figure 1 shows the “drug active ingredient-target” network constructed usingCytoscape software.

#### 3.1.2 Access to Liver-Cancer-Related Targets

HCC-associated targets from the OMIM database and GeneCards database were merged, resulting in a total of 1,067 targets (after duplicates were removed).

#### 3.1.3 Construction of the ZZXJT-Liver Cancer Target Interaction PPI Network

**FIGURE 2:**
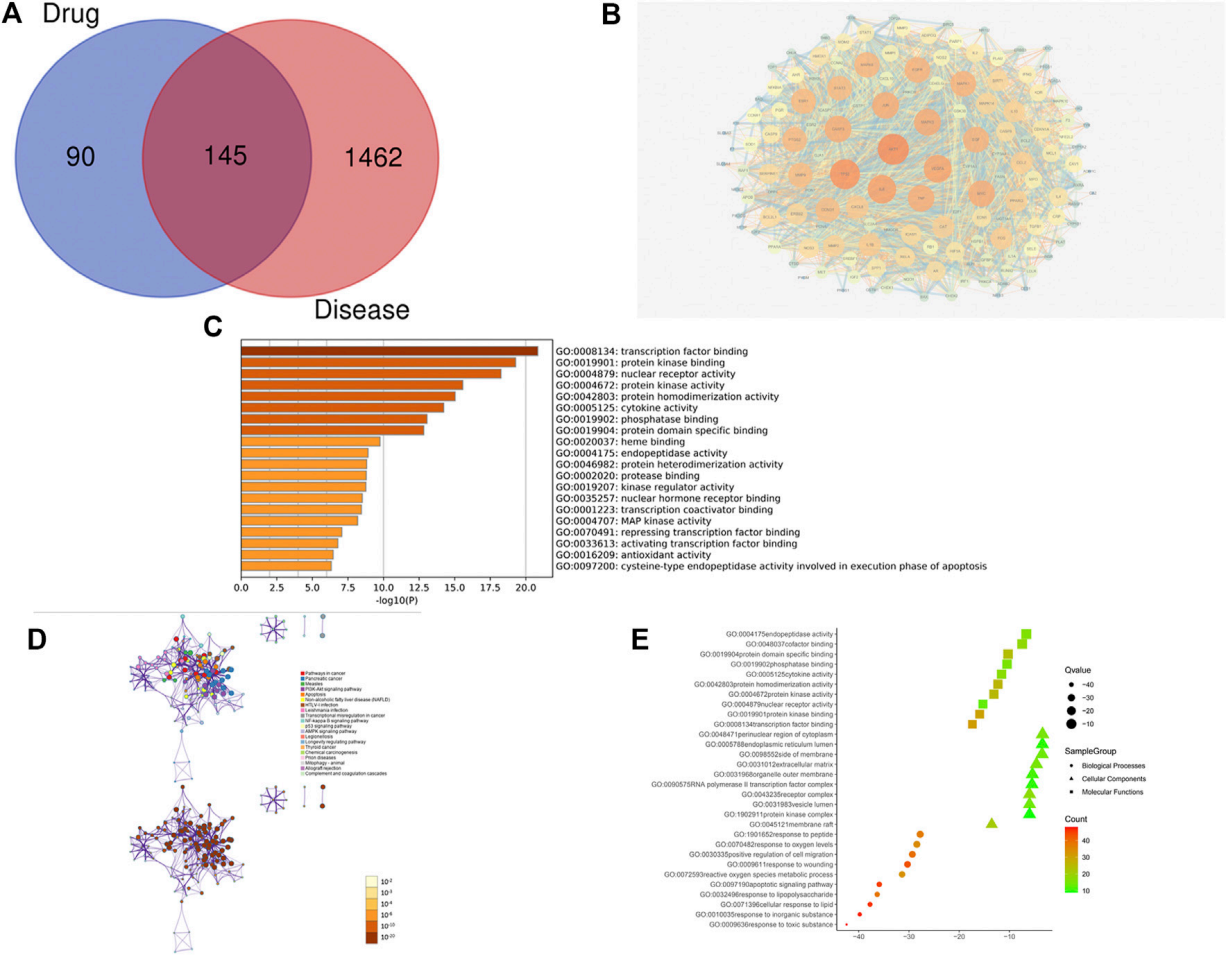
Systems pharmacology model of ZZXJT. Notes: **(A)**: Intersection of ZZXJT predicted targets and liver cancer targets. **(B)**: PPI network of intersection target effect relationship. **(C)**: Main pathways in the systems pharmacology model. **(D)**: Distribution and significance of target proteins in pathway **(C, E)**: The further prediction and analysis of the target’ BP, CC, and MF.

The PPI network from the systems pharmacology modelhas a total of 145 nodes (Figure 2A), which represent predicted targets and 3,167 edges which represent protein-protein interaction relationships (Figure 2B). Darker nodes and larger circles represent greater degree of connectivity. Visualisation revealed that five targets, namely, phosphorylated protein kinase (AKT1), tumour suppressor gene (TP53), interleukin (IL)—6, mitogen activated protein kinase 3 (MAPK3), and vascular endothelial growth factor A (VEGFA) had the highest connectivity scores. This indicates that these five targets were most strongly associated with this model and can be considered as the core five targets of ZZXJTin HCC in the systems pharmacology model.

#### 3.1.4 Enrichment Analysis of Target Pathways and Functions

The enrichment analysis of ZZXJT in relation to HCC targets was conducted using the Metascape data platform, which resulted in 462 KEGG pathways, 755 GO molecular functions, 5670 GO biological processes, and 426 GO cellular components. Only the top 20 results from each group were considered. In the KEGG pathway analysis (*p* < 0.01), pathways related to HCC were PI3K-AKT signalling pathway, apoptosis, and transcriptional misregulation in cancer. Figures 2C,D show the distribution and significance of the proteins involved in each pathway. The exported results for BP, CC, and MF are presented in Figure 2E. BP is mainly involved in the response to toxic substances, response to inorganic substances, cellular response to lipids, response to lipopolysaccharide, and apoptotic signalling pathways. CC is mainly involvedin membrane rafts, vesicle lumens, receptor complexes, protein kinase complexes, and RNA polymerase II transcription factor complexes. MF is mainly involved in transcription factor binding, protein kinase binding, nuclear receptor activity, protein kinase activity, and protein homodimerization activity.

### 3.2 Gene Chip Model of ZZXJT

#### 3.2.1 Chip RNA Quality Control Assessment and Results

**FIGURE 3:**
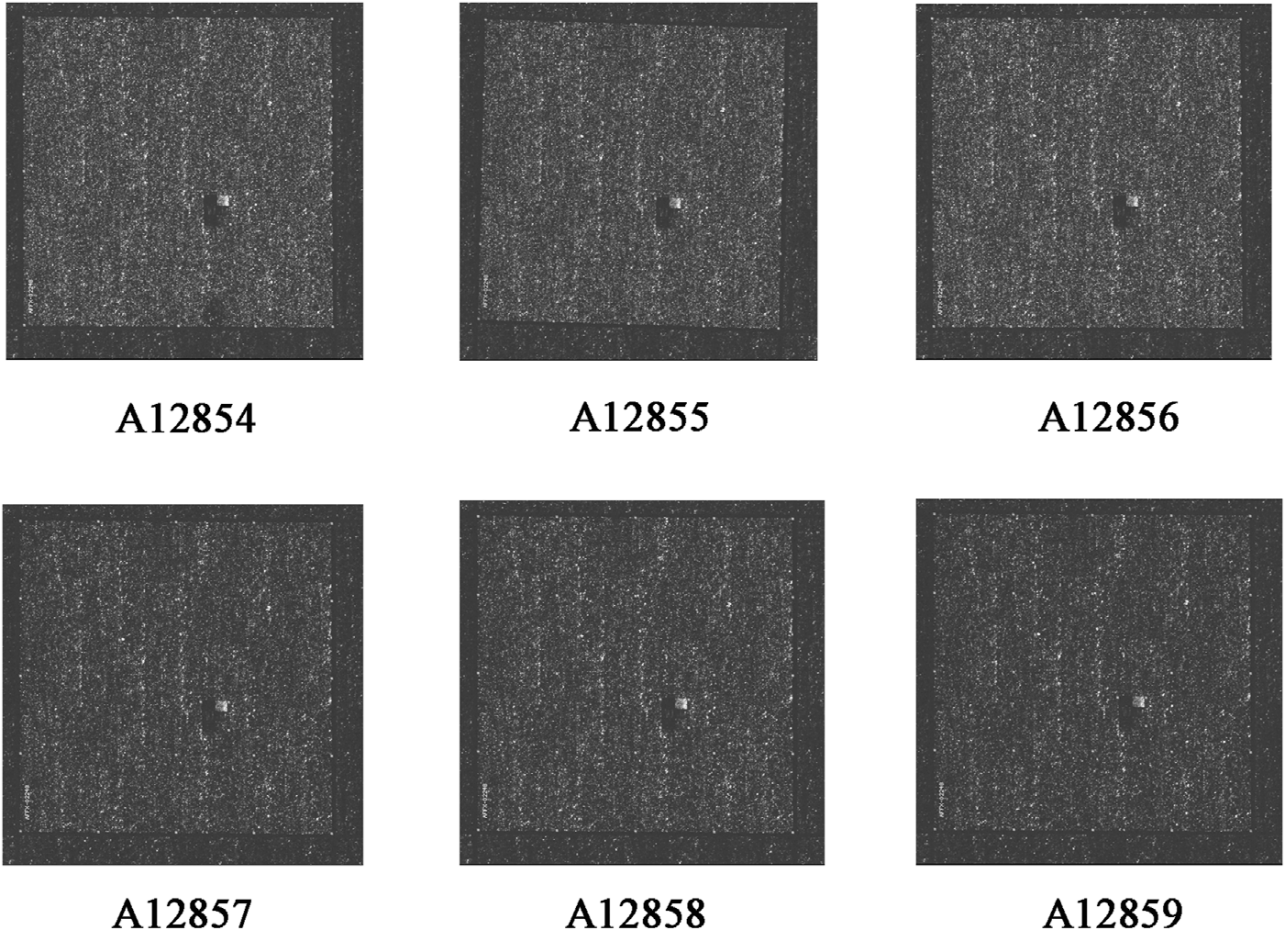
Signal intensity profiles of the samples. Notes: Normal group: A12854, A12855, and A12856; Medication group: A12857, A12858, and A12859.

**FIGURE 4:**
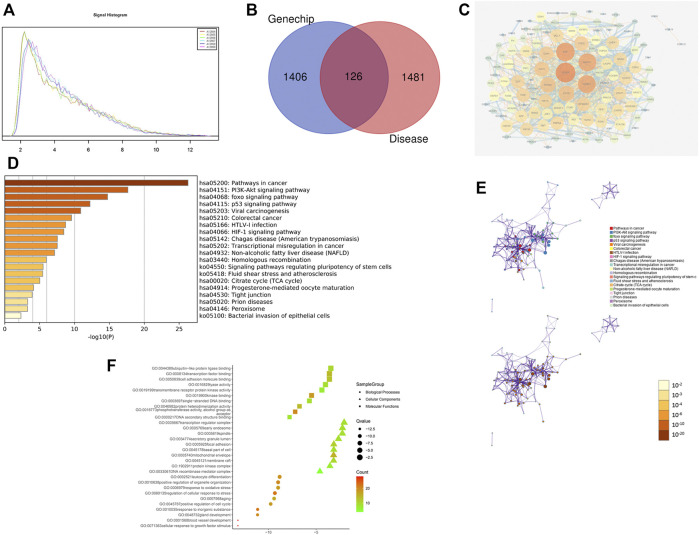
Gene chip model of ZZXJT. Notes: **(A)**: Statistical plot of signal intensity distribution between normal and medication groups. **(B)**: Intersection of ZZXJT predicted targets and HCC targets. **(C)**: PPI network of intersection target action relationship. **(D)**: Main pathways in the gene chip model. **(E)**: Distribution and significance of target proteins in pathway **(D)**. **(F)**: The further prediction and analysis of the target’ BP, CC, and MF.

RNA quality control information of the normal and drug groups was examined using Nanodrop 2000 and Agilent 2,100 (Table 3). Raw data for this experiment were acquired using the GeneChip Scanner 3,000 (Affymetrix, United States) (Figure 3). The signal intensity profiles of the 6 sample sets were calculated. It is assumed that the plots of the signal intensity profiles can demonstrate the signal intensity profiles of all chip probes. As shown in Figure 4A, the abscissa represents the probe signal intensity interval, and the ordinate represents the number of probe sets within the signal intensity interval. The better the sample coincidence of the signal intensity distribution curves, the greater the reliability of the gene chip model. Data was taken for normalisation, resulting in a total of 1,543 differential genes (| fold change | ≥ 2.0 and FDR <0.05), of which 194 were upregulated genes and 1,349 were downregulated genes. The quality control requirements for RNA were metfor subsequent experiments to be conducted and the results of the gene chip analysis were highly reliable and reproducible.

**TABLE 3:** Quality test results.

Sequence	Sample No	Sample name	Thermo NanoDrop 2000		2,100 result		Result
			Concentration (ng/μL)	A260/A280	RIN	28S/18S	
1	A12854	Normal group	2549.6	1.99	9.9	2.4	qualified
2	A12855	Normal group	3077.1	2.00	9.8	2.4	qualified
3	A12856	Normal group	3046.3	1.97	9.7	2.4	qualified
4	A12857	Medication group	2473.3	1.96	9.6	2.5	qualified
5	A12858	Medication group	3035.4	1.99	9.8	2.6	qualified
6	A12859	Medication group	3306.0	1.99	9.8	2.6	qualified

#### 3.2.2 Construction of the ZZXJT-HCC Target PPI Network

A total of 118 nodes with 870 edges representing protein-protein interaction relationships were obtained using Cytoscape software. Darker nodes and larger circles represent a greater degree of connectivity. After visualisation, the greatest connectivity values were observed for the following five targets: vascular endothelial growth factor A (VEGFA), EGF, MAPK1, CCND1, and CYCS. These five targets were most strongly associatedwith this model and can therefore be considered as the core five targets of ZZXJT activityin HCC, as shown in Figures 4B,C.

#### 3.2.3 Enrichment Analysis of Target Pathways and Functions

The enrichment analysis of ZZXJT and HCC-related targets was conducted using the Metascape platform, which resulted in 461 KEGG pathways, 686 GO molecular functions, 5114 GO biological processes, and 484 GOcellular components. Only the top 20 resultsdisplayedby KEGG were considered. In KEGG pathway analysis (*p* < 0.01), pathways related to cancer were the PI3K-AKT pathway, FOXO pathway, p53 pathway, and viral carcinogenesis. Figures 4D,E show the distribution and significance of the proteins involved in each pathway. The export results for BP, CC, and MF are presented in Figure 4F. Each group shows only the top 10 results. BP is mainly involved in blood vessel development, cellular response to growth factor stimulation, glandular development, response to inorganic substances, and positive regulation of the cell cycle. CCmainly involves the DNA recombinase mediator complex, protein kinase complex, membrane raft, mitochondrial envelope, and cell base. MF mainly involves DNA secondary structure binding, phosphotransferase activity, alcohol group as acceptor, protein heterodimerization activity, single-stranded DNA binding, and kinase binding.

#### 3.2.4 qRT-PCR to Detect Changes in the Level of Genes in Cells

**FIGURE 5:**
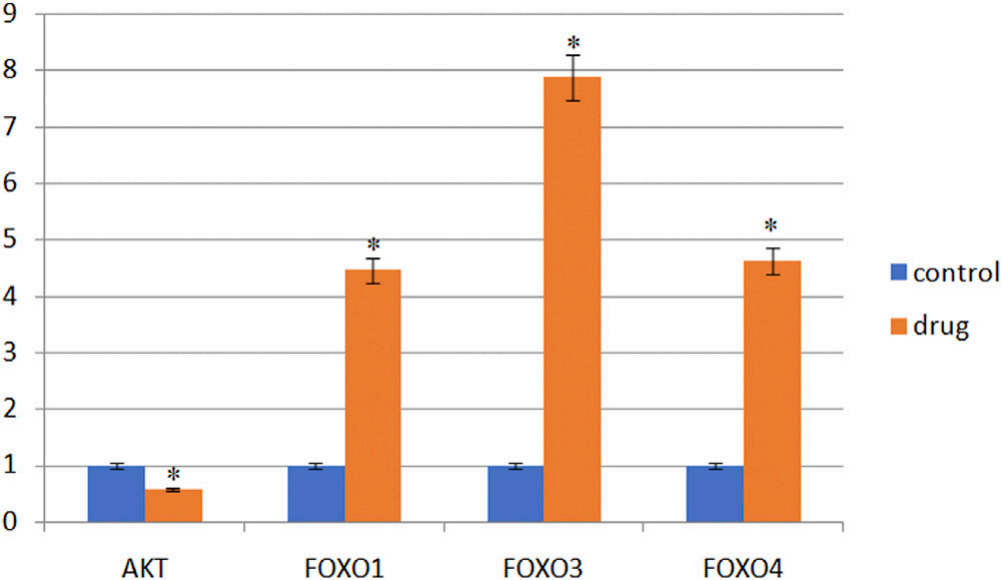
Gene expression in SMMC-7721 cells (‾X ± s). Note: *Compared with the control group, it is statistically significant (*p* < 0.05).

In KEGG pathway analysis, pathways related to cancer were the PI3K-AKT pathway and FOXO pathway. As shown in Figure 5, AKT mRNA expression downregulated compared with the control group (*p* < 0.05). FOXO pathway related genes (FOXO1, FOXO3 and FOXO4) mRNA expression gradually upregulated (*p* < 0.05).

### 3.3 Identity Treatments for Two Models

**FIGURE 6:**
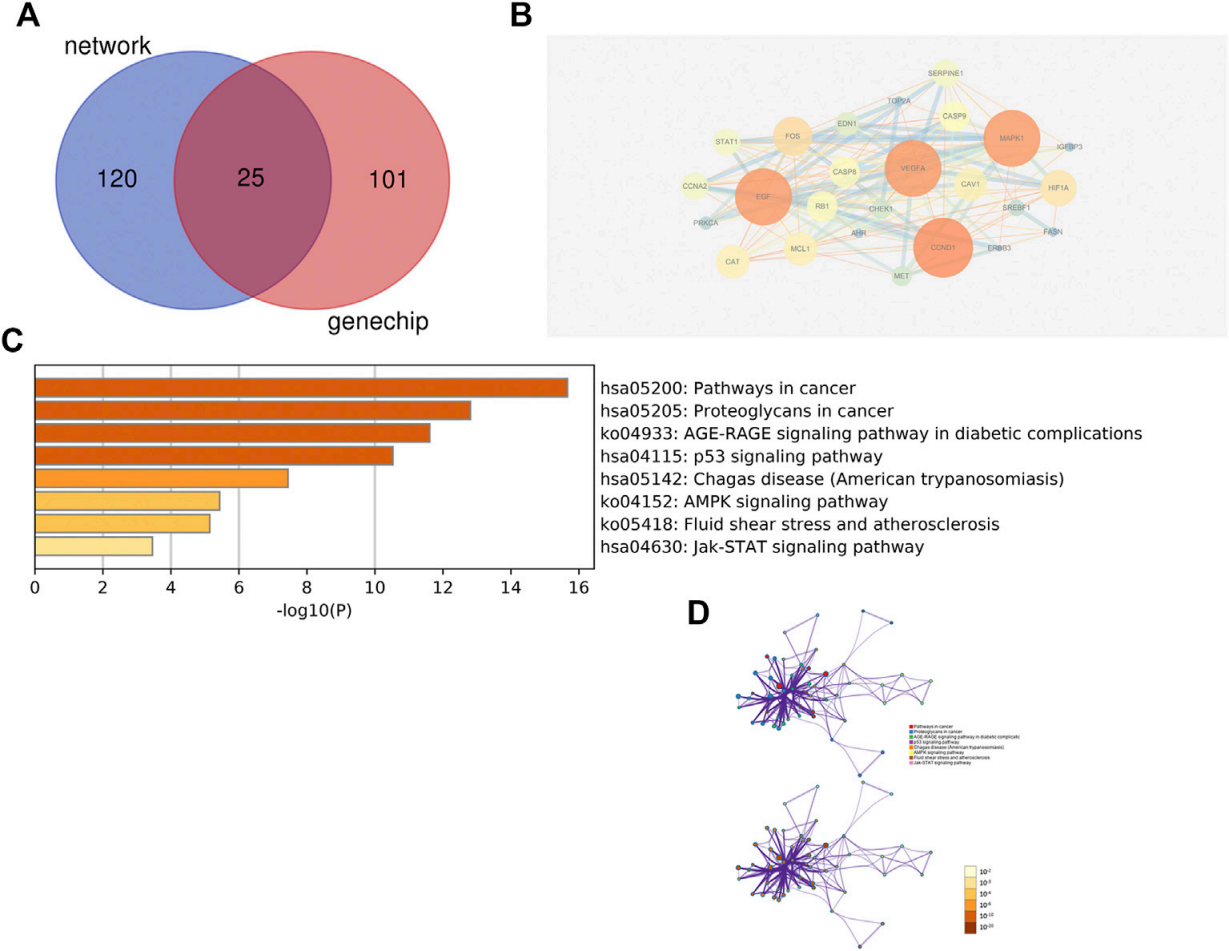
Identical results of systems pharmacology and gene chip model. Notes: **(A)**: The intersection of two models predicted targets and liver cancer targets. **(B)**: PPI network of intersection’s targets. **(C)**: Pathways involved in intersection’s targets. **(D)**: Distribution and significance of target proteins in pathway C.

Using Cytoscape software, a total of 25 nodes with 147 edge lines were obtained. Darker nodes and larger circles represent a greater degree of connectivity. Visualisation results showed that the top five targets, namely, CCND1, vascular endothelial growth factor A (VEGFA), MAPK1, EGF, and FOS, had the highest connectivity values. This indicated that these five targets were the most highly correlated in this model and can therefore be considered as the core five targets for ZZXJT action inHCC in this model. The intersecting targets were further analysed using the Metascape data platform, resulting in eight KEGG pathways. The distribution and significance of the proteins involved in each pathway were analysed. BP, CC, and MF analyses were not performed because of the small number of common targets and the lack of statistical significance of less than 20 pathways (Figure 6).

### 3.4 Molecular Docking Results

#### 3.4.1 Designationof Small Molecules for Docking

A total of 10 small molecules were involved in docking, among which the smallmolecules of the sovereign drug (small molecules common to the drugs were not counted), and the other drugs were numbered EZ1, NZZ1, NZZ2, NZZ3, and NZZ4. The five multidrug common small molecules were numbered A1, A2, A3, B1, and C1.

#### 3.4.2 Collection of Docking Targets

The two models had 25 common targets. One targetprotein structure was removed as it was not searched. The final total of common targets was 24, corresponding to the PDB numbers shown in Table 4. There were three common targets among the top10 target genes of the systems pharmacology model according to “Combined degree”, therefore the targets orderedfrom 1 to 13(PDB numbersshown in Table 5). The top 10 target genes predicted by the gene chip model according to “Combined degree”, included 13 common targets, 2 proteins without crystal structures, and 1 without Homo sapiens protein crystal structure, therefore the number of target genes was ordered from 1 to 26(PDB numbers shown in Table 6).

**TABLE 4:** Common target gene and PDB numbers.

Target gene	PDB No	Target gene	PDB No	Target gene	PDB No
CCND1	2W9F	VEGFA	1VPF	EGF	SL0T
MAPK1	6G54	CCNA2	1OIY	STAT1	3WWT
PRKCA	2GZV	CAT	1QQW	MCL1	6OQC
RB1	7CZG	CASP8	4ZBW	FOS	1FOS
EDN1	1T7H	AHR	5Y7Y	CHEK1	2HOG
MET	3EFJ	TOP2A	5NNE	SERPINE1	3PB1
CASP9	3YGS	CAV1	7LUD	ERBB3	3KEX
SREBF1	1AM9	FASN	2JFD	HIF1A	4H6J

**TABLE 5:** System pharmacology model and PDB numbers.

Target gene	PDB No	Target gene	PDB No	Target gene	PDB No
AKT1	2UZR	JUN	1JUN	TP53	6MY0
TNF	2E7A	IL6	4O9H	MYC	5I50
MAPK3	4QTB	EGFR	3IKA	CASP3	1QX3
MAPK8	4G1W				

**TABLE 6:** Gene chip model and PDB numbers.

Target gene	PDB No	Target gene	PDB No	Target gene	PDB No
CYCS	3NWV	ANXA5	6K25	CD44	1UUH
FGF2	4OEF	RPS6KB1	4L44	NRAS	2N9C
IGF1R	3D94	EZH2	4MI5	APP	4PWQ
HSPA5	3IUC				

#### 3.4.3 Results of Docking

**FIGURE 7:**
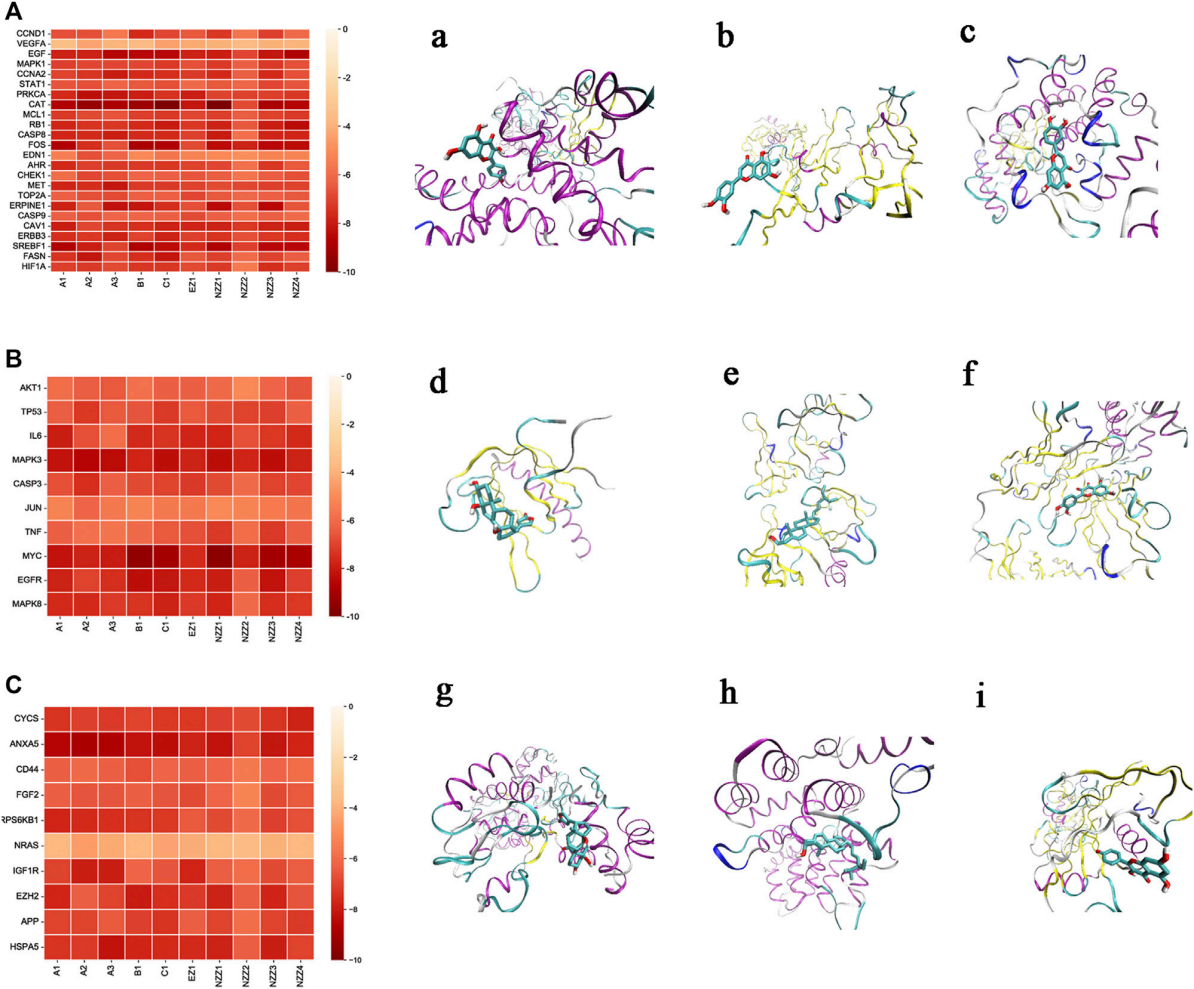
Molecular docking results. Notes: **(A)**: Docking results diagram of common targets and small molecules. **(a)**: The corresponding protein of CCND1 was visualized by docking with small molecule B1 with a docking score of −7.6 kcal mol^−1^. **(b)**: VEGFA corresponds to the protein visualized by docking with small molecule C1 with a docking score of −4.4 kcal mol^−1^. **(c)**: MAPK1 corresponds to the protein visualized by docking with the small molecule C1 with a docking score of −7.2 kcal mol^−1^. **(B)**: Diagram of System Pharmacology model targets and small molecule docking results. **(d)**: The corresponding protein of AKT1 was visualized by docking with the small molecule NZZ4, with a docking score of −6.6 kcal mol^−1^. **(e)**: TP53 corresponding protein was visualized by docking with small molecule A2 with a docking score of −7.3 kcal mol^−1^. f: IL6 corresponds to the protein visualized by docking with small molecule A1 with a docking score of −7.9 kcal mol^−1^. **(C)**: Diagram of docking results of model targets and small molecules of gene chip. **(g)**: CYCS corresponds to the protein visualized by docking with the small molecule NZZ4 with a docking score of −7.8 kcal mol^−1^. **(h)**: ANXA5 corresponds to the protein visualized by docking with small molecule A2 with a docking score of -8.9 kcal mol^−1^. **(i)**: The CD44 counterpart protein was visualized by docking with small molecule B1 with a docking score of −7.9 kcal mol^−1^.

The heatmap (Figure 7) displays the docking resultspresented as free binding energy values (kcal·mol^−1^) ranging from-10 to 0. The 10 small molecules were individually docked with the target protein crystals, and 440 docking results were obtained, comprising 240 for the common target gene proteins and 100 each for the systems pharmacology model and the gene chip model, respectively. VMD software was used to visualise the docked conformation stack plots according to the obtained data to visualise the three optimal strips in each group. The action profiles and visualisations of candidate compounds with each target gene protein are shown in Figure 7 the common target pairsare shown in Figures 7A,a–c, the systems pharmacology target pairs are shown in Figures 7B,d–f, and the gene chip docking targets are shown in Figures 7C,g–i. The three sets of binding free energy results were integrated for statistical comparison, and it was found that there was no statistical difference (*p* > 0.05) in binding free energy for the common target gene proteins (mean = −7.05)and the systems pharmacology model (mean = −7.04). There was a significant statistical difference (*p* < 0.05) in the binding free energy between the common target gene proteins (mean = −7.05) and the genechip model (mean = −6.74).

## 4 Discussion and Conclusion

In this study, the targets of HCC were predictedusing a gene chip model and systems pharmacology model. The results of this paper have been analysed and discussed from the perspective of the contrasts and similarities between the targets predicted by thegene chip and systemspharmacology models, respectively.

It is evident from the results that the system pharmacology approach neglects the relationship between the activity of a drug’s small molecules and the dosage of the drug. Numerous relationshipsbetween small molecules andtargets may be predicted by the systems pharmacology approach, but this method may lead to errors since it simply uses superimposition based on past research results. This error may be augmented in formulation research, which focuses on the relationship between drug dosage, and drug compatibility. The results of this study showed that thecore targetspredicted by systems pharmacology and gene chip accounted for only 17 and 19% of targets, respectively. The similaritiesintargets identified by the two models differed. The top 10 core targets identified by the system pharmacology model only accounted for 2 common targets. Counting the top 10 targets without including the common targets would increase this to 13 targets. The common targets in the top 10 core targets predicted by the gene chip model accounted for 5 targets. Counting the top 10 targets without including the common targets would increase this to 22 targets. Itcan therefore be concluded that the two models were poorly similar based on the target aspect. In terms of KEGG pathway analysis, the top identicalpathwayof the two models was pathways in cancer and the top fiveincluded the PI3K-AKT signalling pathways. While there were common KEGG pathways, except for the inclusion of pathways in cancer, the two modelsdid not share the same pathways, and so the use of KEGG pathway analysis was limited. In conclusion, the gene chip modelis more accurate in target predictions than the systems pharmacology model (Heber and Sick, 2006). Thismay be a direct result of gene chips having been developed as an assay for genetic diseases and as an alternative to clinical disease testing (Huang et al., 2004; Shi et al., 2021). In addition, gene chips usually employ normalisation, which directly expands the target genes that may be involved. In comparison, the predicted results of the systems pharmacology model in this study were poor.

Molecular docking was introduced in this comparative study as a reference prediction model. The binding free energy was used as a benchmark and the top 10 targets from the two models (without co-targets) were individually aligned. Results showed that the docking scores of system pharmacology and co-targets were not statistically significant (*p* > 0.05), while those of genechipsand co-targets were statistically significant (*p* < 0.05). The mean values were larger than the mean values of the common targets. From the docking data of 10 small molecules docked with two model core targets, it can be derived that, because the numerical value is smaller, and the binding is more stable (Hsin et al., 2013). Based on the docking results, system pharmacology was better than the binding of the gene chip. However, experimental results are insufficient to fully reject the prediction that molecular docking is more accurate, possibly due to too little docking data, not only in actual drug effect results but also not in full agreement with binding energy values (Ye et al., 2021). For example, in the vicinity of the target tissue, the lowest drug concentration isa factor that affects the regulation of gene expression (Shin et al., 2020), or when the same drug molecule acts on targets of different cells, and results should differ (Akita and Sliwkowski, 2003). These situations are currently difficult to simulate using a computer model. Although drug absorption was previously modelled through the specification of OB and DL values, such predictions by systems pharmacologymay result in extremely low confidence for a sophisticated and complex network of target structures, especially after multiple error factors are incorporated. Therefore, systems pharmacology corroborated by molecular docking is risky as results may direct researchers incorrectly. Nevertheless, based on the docking data, the predictions of the core small molecules from the two models were consistent, mainly focusing on five small molecules, namely, A1, A2, B1, C1, and NZZ4. Among these, the four consensus small molecules are part of the drug XKC while the remaining four small molecules and A2 are part of NZZ. Based on the concept of“sovereign and adjuvant” in TCM, it is evident that NZZ and XKC play a major role in ZZXJT. Thissupported the TCM theory that adjuvant drugs may also besovereigndrugs. It was inferred that system pharmacology and molecular docking models were of positive significance to identify the main drugs and molecular components in the formula. This was highly consistent with the gene chip.

Systems pharmacology predicts that the targets of the core small molecules are the same, therefore the main treatments should be the same. This prediction in terms of TCM, may wrongly direct researchas important drug-target relationships are circumvented. The integrity of formula research is affected by the simple superposition of a single molecule, which may lead to the low credibility of predicted results. This assumption is made based on systems pharmacology for ZZXJT target prediction of a single formula (ZZXJT), hencemore data is required to firmly establish the reliability of using systems pharmacology predictions in TCM formula research. This can be addressed in future studies.

Since predictions by the systems pharmacology approachare poorly similarto the gene chip technology predictions, we can therefore conclude thatsystems pharmacology has low model confidence and is less reliable. However, the consistency between the core drug predictions versus the core small-molecule predictions was greater in the systems pharmacology model.

## Data Availability

The datasets presented in this study can be found in online repositories, which can be found in the article and Supplementary Material.

## Footnotes

1
https://www.uniprot.org

2
http://www.omim.org

3
https://www.genecards.org

4
http://bioinformatics.psb.ugent.be/webtools/Venn/

5
https://string-db.org

6
http://metascape.org

7
http://www.ehbio.com/

8
http://www.rcsb.org/

## References

[B1] Akita R. W. Sliwkowski M. X. (2003). Preclinical Studies with Erlotinib (Tarceva). Semin. Oncol. 30, 15–24. 10.1016/s0093-7754(03)70011-6 12840797

[B2] Berger S. I. Ma'ayan A. Iyengar R. (2010). Systems Pharmacology of Arrhythmias. Sci. Signal. 3, ra30. 10.1126/scisignal.2000723 20407125 PMC3068558

[B3] Boezio B. Audouze K. Ducrot P. Taboureau O. (2017). Network-based Approaches in Pharmacology. Mol. Inform. 36, 1–11. 10.1002/minf.201700048 28692140

[B4] Cooke E. J. Savage R. S. Wild D. L. (2009). Computational Approaches to the Integration of Gene Expression, ChIP-Chip and Sequence Data in the Inference of Gene Regulatory Networks. Semin. Cel Dev Biol 20, 863–868. 10.1016/j.semcdb.2009.08.004 19682595

[B5] El-Zayat M. M. Eraqi M. M. Alrefai H. El-Khateeb A. Y. Ibrahim M. A. Aljohani H. M. (2021). The Antimicrobial, Antioxidant, and Anticancer Activity of Greenly Synthesized Selenium and Zinc Composite Nanoparticles Using Ephedra Aphylla Extract. Biomolecules 11, 470. 10.3390/biom11030470 33809976 PMC8005055

[B6] Fang J. Cai C. Wang Q. Lin P. Zhao Z. Cheng F. (2017). Systems Pharmacology-Based Discovery of Natural Products for Precision Oncology through Targeting Cancer Mutated Genes. CPT Pharmacometrics Syst. Pharmacol. 6, 177–187. 10.1002/psp4.12172 28294568 PMC5356618

[B7] Heber S. Sick B. (2006). Quality Assessment of Affymetrix GeneChip Data. OMICS 10, 358–368. 10.1089/omi.2006.10.358 17069513

[B8] Hsin K. Y. Ghosh S. Kitano H. (2013). Combining Machine Learning Systems and Multiple Docking Simulation Packages to Improve Docking Prediction Reliability for Network Pharmacology. PLoS One 8, e83922. 10.1371/journal.pone.0083922 24391846 PMC3877102

[B9] Huang H. J. Huang S. L. Lin C. Y. Lin R. W. Chao F. Y. Chen M. Y. (2004). Human Papillomavirus Genotyping by a Polymerase Chain Reaction-Based Genechip Method in Cervical Carcinoma Treated with Neoadjuvant Chemotherapy Plus Radical Surgery. Int. J. Gynecol. Cancer 14, 639–649. 10.1111/j.1048-891X.2004.14418.x 15304160

[B10] Jiao X. Jin X. Ma Y. Yang Y. Li J. Liang L. (2021). A Comprehensive Application: Molecular Docking and Network Pharmacology for the Prediction of Bioactive Constituents and Elucidation of Mechanisms of Action in Component-Based Chinese Medicine. Comput. Biol. Chem. 90, 107402. 10.1016/j.compbiolchem.2020.107402 33338839

[B11] Kou J. P. Yu Z. L. Gong S. Q. Yan Y. Q. (2004). The Actions of Xiaochengqi Decoction, Houpusanwu Decoction and Houpu Dahuang Decoction. Chin. Traditional Patent Med. 2004, 59–61. 10.3969/j.issn.1001-1528.2004.01.020

[B12] Li R. Li Y. Liang X. Yang L. Su M. Lai K. P. (2021). Network Pharmacology and Bioinformatics Analyses Identify Intersection Genes of Niacin and COVID-19 as Potential Therapeutic Targets. Brief Bioinform 22, 1279–1290. 10.1093/bib/bbaa300 33169132 PMC7717147

[B13] Luo C. H. Ma L. L. Liu H. M. Liao W. Xu R. C. Ci Z. M. (2020). Research Progress on Main Symptoms of Novel Coronavirus Pneumonia Improved by Traditional Chinese Medicine. Front. Pharmacol. 11, 556885. 10.3389/fphar.2020.556885 33013395 PMC7516165

[B14] Luo T. T. Lu Y. Yan S. K. Xiao X. Rong X. L. Guo J. (2020). Network Pharmacology in Research of Chinese Medicine Formula: Methodology, Application and Prospective. Chin. J. Integr. Med. 26, 72–80. 10.1007/s11655-019-3064-0 30941682

[B15] Miao S. M. Zhang Q. Bi X. B. Cui J. L. Wang M. L. (2020). A Review of the Phytochemistry and Pharmacological Activities of Ephedra Herb. Chin. J. Nat. Med. 18, 321–344. 10.1016/S1875-5364(20)30040-6 32451091

[B16] Pei L. Bao Y. Liu S. Zheng J. Chen X. (2013). Material Basis of Chinese Herbal Formulas Explored by Combining Pharmacokinetics with Network Pharmacology. PLoS One 8, e57414. 10.1371/journal.pone.0057414 23468985 PMC3585395

[B17] Ren W. Ma Y. Wang R. Liang P. Sun Q. Pu Q. (2020). Research Advance on Qingfei Paidu Decoction in Prescription Principle, Mechanism Analysis and Clinical Application. Front. Pharmacol. 11, 589714. 10.3389/fphar.2020.589714 33584265 PMC7873690

[B18] Ru J. Li P. Wang J. Zhou W. Li B. Huang C. (2014). TCMSP: a Database of Systems Pharmacology for Drug Discovery from Herbal Medicines. J. Cheminform 6, 13. 10.1186/1758-2946-6-13 24735618 PMC4001360

[B19] Shi J. Tao B. Li Z. Song H. Wu J. Qiu B. (2021). Diagnostic Performance of GeneChip for the Rapid Detection of Drug-Resistant Tuberculosis in Different Subgroups of Patients. Infect. Drug Resist. 14, 597–608. 10.2147/IDR.S297725 33633456 PMC7900445

[B20] Shin J. Saini R. K. Oh J. W. (2020). Low Dose Astaxanthin Treatments Trigger the Hormesis of Human Astroglioma Cells by Up-Regulating the Cyclin-dependent Kinase and Down-Regulated the Tumor Suppressor Protein P53. Biomedicines 8, 434. 10.3390/biomedicines8100434 PMC7590133 33086722

[B21] Sun Y. Wu B. Sun M. Wang Y. Che Y. (2017). Expenimental Study of ZhenzhuXiaoji Decoction Inducing Autophagy via STAT3/Survivin Signal Pathway in Hepatoma Cells. Inf. Traditional Chin. Med. 34, 41–44. 10.19656/j.cnki.1002-2406.2017.06.011

[B22] Szklarczyk D. Gable A. L. Lyon D. Junge A. Wyder S. Huerta-Cepas J. (2019). STRING V11: Protein-Protein Association Networks with Increased Coverage, Supporting Functional Discovery in Genome-wide Experimental Datasets. Nucleic Acids Res. 47 (D1), D607–D613. 10.1093/nar/gky1131 30476243 PMC6323986

[B23] Tripathi S. Pohl M. O. Zhou Y. Rodriguez-Frandsen A. Wang G. Stein D. A. (2015). Meta- and Orthogonal Integration of Influenza "OMICs" Data Defines a Role for UBR4 in Virus Budding. Cell Host Microbe 18, 723–735. 10.1016/j.chom.2015.11.002 26651948 PMC4829074

[B24] Trott O. Olson A. J. (2010). AutoDock Vina: Improving the Speed and Accuracy of Docking with a New Scoring Function, Efficient Optimization, and Multithreading. J. Comput. Chem. 31, 455–461. 10.1002/jcc.21334 19499576 PMC3041641

[B25] UniProt Consortium (2021). UniProt: the Universal Protein Knowledgebase in 2021. Nucleic Acids Res. 49 (D1), D480–D489. 10.1093/nar/gkaa1100 33237286 PMC7778908

[B26] Yan Z. Lai Z. Lin J. (2017). Anticancer Properties of Traditional Chinese Medicine. Comb. Chem. High Throughput Screen. 20, 423–429. 10.2174/1386207320666170116141818 28093974

[B27] Yanagawa B. Taylor L. Deisher T. A. Ng R. Schreiner G. F. Triche T. J. (2005). Affymetrix Oligonucleotide Analysis of Gene Expression in the Injured Heart. Methods Mol. Med. 112, 305–320. 10.1385/1-59259-879-x:305 16010026

[B28] Ye M. Luo G. Ye D. She M. Sun N. Lu Y. J. (2021). Network Pharmacology, Molecular Docking Integrated Surface Plasmon Resonance Technology Reveals the Mechanism of Toujie Quwen Granules against Coronavirus Disease 2019 Pneumonia. Phytomedicine 85, 153401. 10.1016/j.phymed.2020.153401 33191068 PMC7837196

[B29] Yuan H. Ma Q. Cui H. Liu G. Zhao X. Li W. (2017). How Can Synergism of Traditional Medicines Benefit from Network Pharmacology? Molecules 22, 1135. 10.3390/molecules22071135 PMC6152294 28686181

[B30] Zhang Y. Wang R. Shi W. Zheng Z. Wang X. Li C. (2021). Antiviral Effect of Fufang Yinhua Jiedu (FFYH) Granules against Influenza A Virus through Regulating the Inflammatory Responses by TLR7/MyD88 Signaling Pathway. J. Ethnopharmacol 275, 114063. 10.1016/j.jep.2021.114063 33813013 PMC9759603

[B31] Zhou Y. Zhou B. Pache L. Chang M. Khodabakhshi A. H. Tanaseichuk O. (2019). Metascape Provides a Biologist-Oriented Resource for the Analysis of Systems-Level Datasets. Nat. Commun. 10, 1523. 10.1038/s41467-019-09234-6 30944313 PMC6447622

